# Supplementary material for: Effect of the FIFA 11+ soccer specific warm up programme on the incidence of injuries: A cluster-randomised controlled trial
Source: PLoS One. 2021 May 24;16(5):e0251839. doi: 10.1371/journal.pone.0251839 (PMC8143390; doi:10.1371/journal.pone.0251839)
Supplement: S1 Checklist — (PDF) [file pone.0251839.s001.pdf]

Strength and limitations analysis of the study using the CONSORT checklist

Section/Topic	*Item No*	*Checklist item*	**Reported on page number**
Title and abstract			
	1a	*Identification as a cluster randomised trial in the title*	Title page
	1b	*Structured summary of trial design, methods, results, and conclusions (for specific guidance see CONSORT for abstracts45 65)*	Abstract, Page 1
**Introduction**			
Background and objectives	*2a*	*Rationale for using a cluster design*	Introduction, Page 3
	2b	*Whether objectives pertain to the cluster level, the individual participant level, or both*	Introduction, Page 4
Methods			
Trial design	*3a*	*Definition of cluster and description of how the design features apply to the clusters*	Methods, Page 5
	3b	*Important changes to methods after trial commencement (such as eligibility criteria), with reasons*	No changes after commencement
Participants	*4a*	*Eligibility criteria for clusters*	Methods, Page 5
	4b	*Settings and locations where the data were collected*	Methods, Page 5
Interventions	*5*	*Whether interventions pertain to the cluster level, the individual participant level, or both*	Methods, Page 6
Outcomes	*6a*	*Whether outcome measures pertain to the cluster level, the individual participant level, or both*	Methods, Page 8
	6b	*Any changes to trial outcomes after the trial commenced, with reasons*	No changes after commencement
Sample size	*7a*	*Method of calculation, number of clusters(s) (and whether equal or unequal cluster sizes are assumed), cluster size, a coefficient of intracluster correlation (ICC or k), and an indication of its uncertainty*	Methods, Page 5
	7b	*When applicable, explanation of any interim analyses and stopping guidelines*	Not Applicable
Randomisation:			
Sequence generation	*8a*	*Method used to generate the random allocation sequence*	Methods, Page 6
	8b	*Details of stratification or matching if used*	Not Applicable
Allocation concealment mechanism	*9*	*Specification that allocation was based on clusters rather than individuals and whether allocation concealment (if any) was at the cluster level, the individual participant level, or both*	Methods, Page 6
Implementation	*10a*	*Who generated the random allocation sequence, who enrolled clusters, and who assigned clusters to interventions*	Methods, Page 6
	10b	*Mechanism by which individual participants were included in clusters for the purposes of the trial (such as complete enumeration, random sampling)*	Methods, Page 6

Section/Topic	Item No	Checklist item	Reported on page number
	10c	*From whom consent was sought (representatives of the cluster, or individual cluster members, or both) and whether consent was sought before or after randomisation*	Methods, Page 5
Blinding	*11a*	*If done, who was blinded after assignment to interventions (for example, participants, care providers, those assessing outcomes) and how*	Methods, Page 8
	11b	*If relevant, description of the similarity of interventions*	Not Applicable
Statistical methods	*12a*	*How clustering was taken into account outcomes*	Methods, Page 8
	12b	*Methods for additional analyses, such as subgroup analyses and adjusted analyses*	Methods, Page 8
Results			
Participant flow (a diagram is strongly recommended)	*13a*	*For each group, the numbers of clusters that were randomly assigned, received intended treatment, and were analysed for the primary outcome*	Results, Page 9
	13b	*For each group, losses and exclusions for both clusters and individual cluster members*	Results, Page 9
Recruitment	*14a*	*Dates defining the periods of recruitment and follow-up*	Results, Page 9
	14b	*Why the trial ended or was stopped*	Not applicable
Baseline data	*15*	*Baseline characteristics for the individual and cluster levels as applicable for each group*	Results, Page 9-10
Numbers analysed	*16*	*For each group, number of clusters included in each analysis*	Results, Page 9-10
Outcomes and estimation	*17a*	*Results at the individual or cluster level as applicable and a coefficient of intracluster correlation (ICC or k) for each primary outcome*	Results, Page 11
	17b	*For binary outcomes, presentation of both absolute and relative effect sizes is recommended*	Results, Page 12-14
Ancillary analyses	*18*	*Results of any other analyses performed, including subgroup analyses and adjusted analyses, distinguishing*	Results, Page 12-14
Harms	*19*	*All important harms or unintended effects in each group (for specific guidance see CONSORT for harms42)*	Discussion, Page 15
Discussion			
Limitations	*20*	*Trial limitations, addressing sources of potential bias, imprecision, and, if relevant, multiplicity of analyses*	Discussion, Page 20
Generalisability	*21*	*Generalisability to clusters and/or individual participants (as relevant)*	Conclusion, Page 21
Interpretation	*22*	*Interpretation consistent with results, balancing benefits and harms, and considering other relevant evidence*	Discussion, Page 15-19
Other information			
Registration	*23*	*Registration number and name of trial registry*	Methods, Page 5
Protocol	*24*	*Where the full trial protocol can be accessed, if available*	Not Applicable
Funding	*25*	*Sources of funding and other support (such as supply of drugs), role of funders*	Not applicable
