# Supplementary material for: Effect of the FIFA 11+ soccer specific warm up programme on the incidence of injuries: A cluster-randomised controlled trial
Source: PLoS One. 2021 May 24;16(5):e0251839. doi: 10.1371/journal.pone.0251839 (PMC8143390; doi:10.1371/journal.pone.0251839)
Supplement: S1 Protocol — (PDF) [file pone.0251839.s002.pdf]

SOCCER INJURY SURVEILLANCE AND IMPLEMENTATION OF AN INJURY PREVENTION PROGRAMME IN RWANDA

Author: AssumanNuhu

Supervisor: Dr. Theresa Burgess

Co-supervisor: Prof. Jennifer Jelsma

TABLE OFCONTENTS

LIST OF FIGURES iii

LIST OF TABLES iv

LIST OF APPENDICES v

GLOSSARY OF TERMS vi

CHAPTER ONE: INTRODUCTION 1

1.1 BACKGROUND OF THE STUDY 1

1.2 STATEMENT OF THE PROBLEM 4

1.3 AIMS AND OBJECTIVES OF THE STUDY 5

CHAPTER TWO: METHODOLOGY 7

2.1 RESEARCH SETTING 7

2.2 ORGANISATION OF THE STUDY 8

2.3STUDY ONE: INTRODUCTION OF INJURY SURVEILLANCE SYSTEM 10

2.3.1Aim 10

2.3.2Objectives 10

2.3.3 Study design 10

2.3.4 Participants 10

2.3.5 Data collection instruments 12

2.3.6 Procedure 14

2.3.7 Pilot study 16

2.3.8 Data management 17

2.3.9 Statistical analysis 17

2.3.10 Ethical considerations 18

2.4STUDY TWO: INJURY PREVENTION INTERVENTION 18

2.4.1 Aim 1 18

2.4.2 Aim 2 18

2.4.3 Study design 19

2.4.4 Participants 19

2.4.5 Pre- and post-tests 21

2.4.6 Intervention 23

2.4.7 Procedure 24

2.4.8 Statistical analysis 27
2.4.9 Ethical considerations 28
REFERENCES 29

LIST OF FIGURES

Figure 1: Haddon Intervention Matrix with Examples of Sport Injury Prevention Measures 2
Figure 2: Study one framework 16
Figure 3: Study two framework 26

LIST OF TABLES

Table 1: The TRIPP framework for research leading to real-world sports injury prevention……9

LIST OF APPENDICES

Appendix A : LIST OF PLAYERS' FORM
Appendix B: MEDICAL RECORD FORM
Appendix C: PLAYER EXPOSURE FORM
Appendix D: DAILY INJURY REPORT FOR FOOTBALL INJURIES
Appendix E: DESCRIPTION OF THE TEST TO BE PERFORMED
Appendix F: COACHES' INFORMATION SHEET
Appendix G: PLAYERS' INFORMATION SHEET
Appendix H: INFORMATION SHEET FOR PLAYERS' PARENTS OR LEGAL GUARDIANS
Appendix I: INFORMED CONSENT FORM
Appendix J: DATA ANALYSIS TABLE
Appendix K: QUESTIONNAIRE FOR COACHES
Appendix L: 11+ WARM UP EXERCISES
Appendix M: CONTENT FOR THE TRAINING
Appendix N: THE "11+" RECORDING FORM

GLOSSARY OF TERMS

Coach: One who instructs players in the fundamentals of a competitive sport and directs team strategy (Merriam-Webster Online Dictionary, 2010).

Exposure factor: Individual participation time recorded (Hägglund, Waldén, & Ekstrand, 2006)

Recurrent injury: injury of the same type and at the same site which occurs after a player's return to full participation within two months (Fuller et al., 2006).

Injury prevention: the implementation of interventions to eliminate or reduce the likelihood and the severity of injuries caused by external or internal mechanisms before they occur (Lawrence, 2008).

Injury severity: Based on absence from game or practice participation: slight (1–3 days), minor (4–7 days), moderate (8–28 days), major (>28 days) (Fuller et al., 2006).

Rehabilitation: A player is considered injured until given clearance by medical staff to participate fully in team training and match play (Hägglund et al., 2006).

Soccer Injury: Any physical complaint resulted for soccer participation whether in training or match (Junge & Dvorac, 2000).

Training session: Coach directed physical activities (on field, gym, recovery sessions, aerobics) carried out with the team (Hägglund et al., 2006).

Readiness for injury prevention: Coach related factor that is assumed to influence sports injury outcomes (Siwon, 2013).

CHAPTER 1: INTRODUCTION

1.1 BACKGROUND OF THE STUDY

The practice of soccer predisposes players to encounter an increased number of injuries over time due to the high intensity nature of the game and contacts (Alentorn-Geli et al., 2014). Many studies have indicated that the lower extremities are the parts of the body that are most frequently affected by injuries. Some have shown that the ankle joint was the most affected part (Luciano & Lara, 2012), others the knee joint (Alentorn-Geli et al., 2014) and others the thigh (Ekstrand, Hägglund, & Waldén, 2011).

Since soccer is a contact game, many injuries are considered to be of traumatic nature where ankle and knee sprains were observed to be the most encountered injuries (Nilstad, Andersen, Bahr, Holme, & Steffen, 2014). A recent study conducted in Brazil indicated that most injuries were muscular and most occurred as the result of collisions with other athletes (de Freitas Guina Fachina et al., 2013). In other studies, the lower back, Achilles tendon, groin, patellar tendon, and lower leg are the typical sites of overuse injury observed among soccer players (Kristenson et al., 2013).

Junge and Dvorak (2004) concluded that the nature and severity of injuries hamper the performance of players and that every soccer player participating in high level competition sustains at least one injury that will hinder the performance. There is significant medical and economic cost associated with sports injury affecting athletes, teams and society. Sports injuries were estimated directly to cost the Australian community at least $1.65 billion 1998 (Orchard & Finch, 2002). Looking at the situation in Rwanda where players and teams have economic difficulties, soccer players might have trouble managing their injuries. However, few reports have been done to ascertain the burden of soccer injuries in terms of cost.

Suggestions have been made to counter the occurrence of injuries. In accordance with the framework developed by Van Mechelen, Hlobil, and Kemper, (1992) and later upgraded by Finch (Finch, 2006) preventive measures should be based on epidemiological research. The first two steps in injury prevention in a given sporting activity should be the establishment of the extent, incidence, severity and profile of injury. In Rwanda, a few systematic epidemiological studies looking at soccer-related injuries were conducted that focus on the type, severity, and management of injuries, the need for physiotherapy intervention (Twizere, 2004) among male

players, and factors associated with sports injuries among female soccer players (Niyonsenga & Phillips, 2013). Although these studies used different methods of data collection and the definition of injury, they highlighted that the number of injuries were high and recommended injury prevention intervention.

From a public health perspective, prevention of injuries should consider all the factors that constitute a chain of events leading to injuries. Therefore injury prevention programmes should be designed to help players avoid incidents ('pre-event' measures), to avoid immediate severity of injury in case an incident happens ('event' measures),or to minimise the consequences of a sustained injury ('post-event' measures) (Henke & Luig, 2012). These steps could be further summarized as primary, secondary or tertiary levels of injury prevention. Henke and Luig, (2012) summarized injury prevention measures using the Haddon-injury prevention matrix (Figure 1) following the comprehensive injury causation model developed by Bahr and Krosshaug (2005) indicating all the intrinsic and extrinsic factors predisposing players to injuries.

Figure 1: Haddon Intervention Matrix with Examples of Sport Injury Prevention Measures

(Henke & Luig, 2012)

		FACTORS		
Phase		Human	Equipment	Environment
Pre-Accident	Accident prevention	Risk awareness<br>Pre-season conditioning<br>Strength training<br>Warm-up	Braces<br>Proper foot-ware<br>Playing field surface	Fair play rules<br>Playing field management<br>Safety rules and regulations
Injury-event	Injury-prevention	Fall techniques<br>Impact coping techniques	Skin guards<br>Mouth protection<br>Face guards	Padded goal posts<br>Shock absorbing surfaces
Post-accident Event	Injury treatment and rehabilitation	Fair training<br>Compliance to “return-to-play” rules	First aid equipment<br>Emergency equipment	Emergency and rescue services<br>Medical care and rehabilitation services

Many studies that have been conducted in soccer assessing the effectiveness of injury prevention focused mainly on the primary (Junge et al., 2011) and tertiary injury prevention strategies. However, there are few studies that focused on the secondary injury prevention. Therefore there is a need for more studies to investigate the effectiveness of strategies targeting all the levels to prevent injuries in soccer. For example, a study conducted by Mandelbaum et al.(2005) assessed the benefits of performing neuromuscular and proprioceptive exercises on the reduction of knee injuries with emphasis to the Anterior Cruciate ligament. Another study tested the effect of a stretching technique on the occurrence of hamstring strain among elite soccer players (Arnason, Andersen, Holme, Engebretsen, & Bahr, 2008). Many other research attempts were conducted looking at the effectiveness of other techniques in injury prevention. Even though studies vary in many areas such as the population, the designs, and the intervention used, they conclude that soccer injuries can be prevented and suggest that multi-component intervention studies would be effective.

Following proposed multiple factor intervention strategies, recent studies were conducted using the model developed by the F-MARC. The F-MARC developed an injury prevention programme called the "11+" composed of structured exercises performed during warm up plus the promotion of fair play. The results of studies conducted using the "11+" programme show the reduction in the risk of injuries (RR 0.65; 95% CI 0.46 to 0.91) (Soligard et al., 2010), severe (RR 0.47; 95% CI 0.26to0.85) as well as overuse injuries (RR 0.55; 95% CI 0.36 to 0.83) (Soligard et al., 2008). Additionally, other studies highlighted that the programme increased proprioception, static and dynamic balance (Daneshjoo, Mokhtar, Rahnama, & Yusof, 2013), functional balance (Kathrin Steffen et al., 2013) and concentric hamstring strength (Daneshjoo et al., 2013). However, the results of other studies do not indicate significant effect of the "11+" programme (van Beijsterveldt et al., 2012; Steffen, Myklebust, Olsen, Holme, & Bahr, 2008). The major limitation for these studies was lack of coaches and players' compliance in the implementation of the programme. Therefore there is a need to further investigate the evidence on the effectiveness of the "11+" programme on the prevention of injuries in soccer.

Studies that investigated the second level of injury prevention are scarce. Correspondingly the F-MARC developed the guideline that could be used by soccer people to decide if the player could continue playing in case of injury. However, many injuries could be aggravated when the players

are allowed to resume training or competition without any sort of care or appropriate assessment. Therefore there is a need for an interventional study that focuses on the outcome of implementation of first aid and return to activities in teams that do not have any medical personnel.

According to Hägglund, Waldén and Ekstrand, (2007) players in the teams without any medical personnel have difficulties to get appropriate treatment and rehabilitation of their injuries. The authors conducted a study involving coaches on return to play decision of injured players among teams that did not have any medical personnel. The results indicate that the rate of sustaining a re-injury was lower among the teams after the intervention. Moreover, there is a need to assess the incidence of the re-injuries among team that have and teams that do not have medical personnel.

The role of the coach is valuable in maintaining health, safety and success of the players (Sharkey & Gaskill, 2006). While supervising players during training and competition, the coach should recognize potentially risky situations and develop strategies to minimize them. On the contrary, often coaches are not equipped with the necessary knowledge for them to identify the risk of injuries, treatment, rehabilitation and the modalities for prevention (Andersen, Courson, Kleiner, & McLoda, 2002). However, little is known about what coaches know regarding injury prevention. It would be of great importance to assess the level of knowledge of the coaches in first aid and injury prevention. Furthermore, they should be trained in emergency and first aid to improve their knowledge.

1.2 STATEMENT OF THE PROBLEM

In Rwanda a large number of young people participate in soccer at different levels. Players incur injuries as they increase their involvement in trainings and matches. As mentioned by Junge and Dvorak (2004) players sustain performance limiting injuries every year. Players in Rwanda may have serious problems with their performance that may be due to the severity of injuries and extended period of time without playing. Therefore many players may not have the opportunity to proceed to high performance and top level play. The development of the young players may also be affected as a result of injury where psychological and behavioural problems were displayed (Verhagen, van Stralen, & van Mechelen, 2010).

Injury prevention has been advocated to help players to stay injury-free by proper training, warm up, cool down, stretching and adequate recovery (Kirkendall, Junge, & Dvorak, 2010; Colin Fuller, Junge, & Dvorak, 2012). Additionally a study conducted in Rwanda shows the need for preventive measures as soccer players are threatened by the reduction of their performance and stop their careers at an early stage due to injuries (Twizere, 2004). Coaches and players should be aware and take the lead to minimize the risk for injuries and follow appropriate treatment and rehabilitation principles. However, coaches and players may have deficits in the knowledge and implementation of injury prevention measures. Moreover, teams may be experiencing insufficient financial resources and shortage of medical personnel. There is a need for injury prevention intervention programmes targeting coaches to address injuries in youth soccer in Rwanda.

1.3 AIMS AND OBJECTIVES OF THE STUDY

Study one

AIM

The purpose of the study is to establish an injury surveillance system in soccer teams in Rwanda.

Objectives

a. To determine the difference in etiology, nature, severity and location of injuries between first and second division soccer players
b. To assess whether there is any relationship between injury pattern and players' age, playing position and division of play.
c. To determine whether nonmodifiable and modifiable intrinsic risk factors predict the risk of sustaining an injury in male soccer players in Rwanda.
d. To assess if there is any difference in injury pattern according to training and match exposure
e. To determine whether the availability of the medical support into the teams is associated with the incidence of re-injuries

Study two

AIM 1

One purpose of this study is to explore the difference in the coaches' knowledge, perception of, and readiness to accept a sports injury prevention programme.

Objectives

a. To assess if there is any difference in the knowledge of coaches towards injury prevention before and after the intervention
b. To assess if there is any difference in perception of the soccer coaches towards injury prevention before and after intervention
c. To assess whether the introduction of an injury prevention programme will change the readiness[1] for injury prevention practice of soccer coaches

AIM 2

A second purpose of the study is to assess the use of a warm up programme on the reduction of injuries in soccer.

Objectives

a. To assess if there is any difference in the occurrence of injuries before and after the introduction of the injury prevention warm up programme
b. To assess if the compliance of the injury prevention warm up programme will be associated with the reduction injuries.
c. To determine if there is a difference in injury incidence between the intervention groups and the control group

[1] Readiness for injury prevention practice is defined as the factor that persuades coaches' engagement in activities that will influence soccer injury outcome (Siwon, 2013).

CHAPTER 2: METHODOLOGY

2.1 RESEARCH SETTING

The study will be conducted in Rwanda which is a land locked country situated in central Africa with a surface area of 26,338 km^2 (www.gov.rw). Rwanda is bordered to the North by Uganda, to the East by Tanzania, the Democratic Republic of Congo to the West and Burundi to the South. The population of Rwanda is 12 million with 54% of the population being under 19 years of age (EICV3). This is seen as a huge opportunity by the government of Rwanda to develop talent and the sport of soccer specifically.

The performance of Rwanda in sports in the past decade has improved because of the interest and investment of the government and the population in different aspects. In its quest to be recognized a 'sporting' nation the government of Rwanda tries to fairly support all the sports disciplines. There are approximately 26 national sports federations in Rwanda. Some of them have gained international recognition namely football (Soccer), paralympics, athletics, cycling, basketball and volleyball.

Currently, the soccer governing body, the Federation of Rwandan Football Association (FERWAFA) is a member of the African Football Confederation and has been affiliated with the International Federation of Football Association since January 1976 (FIFA, 2014).Each year clubs from Rwanda national teams are called upon to participate in tournaments that are organized by the continental and world governing bodies. There are 14 clubs competing in the top or first division, 19 clubs in the second division in the male category while 10 clubs participate in the female category. There are other soccer competitions organized at primary, secondary and university levels which also involve the participation of a great number of young sports men and women which will not be the focus of this study.

2.2 ORGANISATION OF THE STUDY

This study is divided into two parts: one study (study 1) is a prospective cohort study that will focus on the introduction of an injury surveillance system in soccer clubs while the other part (study 2) is a randomised controlled trial that will be conducted among teams in the second division.

Both studies will be based on the Translating Research into Injury Prevention Practice framework (TRIPP) proposed by (Finch, 2006). The framework has six steps that need to be followed to build the evidence based prevention intervention (Table 1). The first two steps propose the introduction of injury surveillance and the establishment of the etiology and mechanisms of injuries. This is in line with the part one of this study which aims at establishing a system of monitoring injuries among soccer players. During this phase, additional information pertaining to the severity, burden, and treatment of injuries will be sought. Additionally, players and coaches characteristics will be assessed as well as the medical assistance available into the team.

The third step of the framework proposes the development of injury prevention measures. Literature has shown the effectiveness of a number of injury prevention strategies. The recommendation arising from recent literature is the use of an injury prevention programme called the "11+" that targets multiple factors. It is composed of 15 exercises that are divided into three parts. Part one includes running exercises at a low speed combined with active stretching and controlled partner contact. Part two is composed of six sets of exercises focusing on core and leg strength, balance and plyometrics/ agility, each with three levels of increasing difficulty. Part three is composed of running exercises at a moderate/ high speed combined with planting/ cutting movements. The exercises are designed to be incorporated into the warm up programme.

The fourth step of the framework proposes the use of scientific evaluation of the developed programme. This step corresponds to the second part of this study where the programme will be

introduced to the coaches. Additional training will be provided to the coaches of the teams in the control group pertaining to first aid and injury treatment, rehabilitation. It is believed that this study will take into consideration the knowledge and uptake of the introduced injury prevention measures. At the end of this study, injury prevention context will be identified that will help to propose a well tailored countrywide intervention as described in the stage five and six of the TRIPP framework.

Table 1: The TRIPP framework for research leading to real-world sports injury prevention

**STAGE**	**MODEL**	**Scope of the study**
1	Injury surveillance	**Study 1**<br>Injury surveillance system
2	Establish etiology and mechanism of injury	
3	Develop preventive measures	**Study 2**<br>Comprehensive primary, secondary and tertiary prevention intervention
4	Ideal condition / Scientific evaluation	
5	Describe intervention context to inform implementation strategies	Conclusions and recommendations
6	Evaluate effectiveness of preventive measures in implementation context	Dissemination

(Finch, 2006)

2.3STUDY ONE: INTRODUCTION OF INJURY SURVEILLANCE SYSTEM

2.3.1Aim

The purpose of the study is to establish an injury surveillance system in soccer teams in Rwanda.

2.3.2Objectives

a. To determine the difference in etiology, nature, severity and location of injuries between first and second division soccer players
b. To assess whether there is any relationship between injury pattern and players' age, playing position and division of play.
c. To assess if there is any difference in injury pattern according to training and match exposure
d. To describe the availability of the medical support into the teams

2.3.3 Study design

A prospective cohort study design will be used to follow up players for a period of two consecutive seasons.

2.3.4 Participants

The Federation of Rwandese Football Association (FERWAFA) organizes tournaments at different levels. The top flight male first division is composed of 14 teams while 19 clubs compete in the second division.

The first division competition is the country's primary soccer competition. There are 14 men's association soccer clubs that participate in the league and it operate on the process of promotion and relegation. The teams play 26 matches, where each club plays the others twice, during the season which runs from September to June. The total number of matches is 364 matches. The league is currently sponsored by the largest brewer and soft beverage company in Rwanda on its band new beer named "turbo king" and thus officially known as "turbo king national football league". At the end of the completion, the teams are ranked according to the total points accumulated during the season. In case of equal number of points the goal calculation of the number of goals scored and conceded come in favor of the team to determine the winner. The

league winner qualifies to represent the country in the CAF champions' league while the two lowest placed teams are relegated into the second division. Many teams in the first division are sponsored either by private business companies or government institutions (for example, the police, the army and other governmental parastatals) (FERWAFA, 2008).

There are 19 teams competing in the second division organized by the FERWAFA. Unlike the first division, the second division teams do not play each other because each team would play 36 games. Playing many games require many resources from the teams, the organizing federation as well as a longer period of time. Therefore the teams are randomly divided into two groups. Group A composed of 10 teams and group B composed of 9 teams. Teams in each group play each other and accumulate the points according to the win or draw rules. The first two teams in each group qualify to the semifinal stage where they play the teams from the other group. The winners of the semifinal stage qualify to the final and are automatically promoted to the fist division. The competition ends while the teams play between 11 and 12 matches. The second division season runs from November to May. The FERWAFA requires each team to register maximum 30 players; therefore the maximum number of players that will participate in this study are 990.

Inclusion criteria

Players who will be having contracts with the teams will be included in this study. Prior to the start of the league players undergo a medical assessment to be cleared and issued a playing license. Therefore, players having a valid license to participate in the league from the FERWAFA will be included. Players presenting injury at the beginning of the study will be included when cleared to join teams' training and matches. Data from players who will be selected to participate in the national team will also be included in the study.

Exclusion criteria

Players who will not agree to participate in the study will be excluded from the study.

2.3.5 Data collection instruments

2.3.5.1 List of the players

The list of all the players will be obtained from the FERWAFA (Appendix A). It will be sent to the teams while additional information pertaining to age, playing position, number of years playing soccer and number of year in the current team will be needed.

2.3.5.2 Medical record form

Additionally, the medical record form in terms of questionnaire (Appendix B) will also be sent to the players to screen information pertaining to the past medical history of the players. The history of injury would be assessed including the number of injuries sustained, causes, mechanisms, body part affected, type as well as the treatment received.

2.3.5.3 Exposure form

A specific player exposure form developed by FIFA will be used to record detailed information regarding player involvement in soccer (Fuller et al., 2006) (Appendix C). The forms will be given to the coaches to record duration of the training sessions. Match exposure will be gathered from the referees' reports submitted to FERWAFA. Any form of physical activities supervised by the coach that the players will undertake during training will be recorded as training exposure time. Match exposure time is defined as the amount of time the player will be involved while the team is playing against another team. The duration of the training will be recorded from the start of the warm up to the end of the cool down session (Parry & Drust, 2006). Exposure form will also record player absence from training and competition and the reason for this absence The forms will be collected by the research team once in two weeks.

2.3.5.4 Injury registration form

Similar to the exposure form, an injury report form (Appendix D) will be used to collect data pertaining to the incidence of injuries by the team medical personnel or the research assistant for

the teams that do not have any medical personnel (Fuller et al., 2006). The form is comprehensive and designed in such a way that the injuries are recorded on the front side of the page while all the definitions pertaining to the type, anatomical location, severity and circumstance of injury are provided at the back page of the form. The form has been developed and used during major FIFA tournaments and Olympic games (Junge, 2004) and has been extensively used in other studies (Junge et al., 2011) but is not routinely used in soccer in Rwanda. For example an injury is defined as any physical complaint that resulted from soccer participation whether in training or match. It does not necessarily mean that there is a need or not of medical attention.

A meeting with the team medical personnel will be organized to explain to them about the purpose of the study and their role in the study. They will be given the report forms to record any injury that will happen during the time of the study. Further explanations will be given to them as to specific definitions and the way of recording.

2.3.5.5 Assessment of the soccer players

A number of musculoskeletal assessments will be conducted in order to establish intrinsic risk factors for injuries (Maffey & Emery, 2006; Brumitt et al., 2013; Bahr & Krosshaug, 2005). A number of assessment techniques will be performed do identify weakness, limitation, or impairment that could be associated with the occurrence of injuries. The assessment techniques include the anthropometric assessment, the range of motion and flexibility of muscles, the specific neural mobility tests, stability, strength as well as proprioception testing. Due to minimal resources, equipment and time, on-field testing will be performed. The field will be set up in a circuit form where each circuit will be monitored by the research team members. Each station will have the description of the procedure to ensure that all the steps are followed and all the subjects are undergoing the same amount of difficulties. Though the research assistants will be trained about the measurements, three readings will be used and average will be recorded or as directed by the test protocol. Each test and measurements are described in the appendix E.

2.3.6 Procedure

2.3.6.1 Data collection

Ethical approval for the study will be sought from the University of Cape Town. Permission to conduct the study will be requested from the ministry of sports and culture, and FERWAFA. Finally, permission will be sought from the teams in the first and second division. Written consent will be obtained from the coaches and players from both divisions as well as for the parents or legal guardians of under 18 players (Appendices F, G, H, I).

Physiotherapists with Bachelor's degree will be recruited as research assistants. Training will be conducted where they will be informed about the research aims, objectives and the significance of the study. They will be trained about the methods of data collection of the study. They will be taught about the ethical considerations as well as their role in the study. They will further be familiarized with the tools that will be used for data collection to ensure reliability and standardization among the data collectors. The training will be done using the guidelines for collecting data in studies of soccer developed by the FIFA (Fuller et al., 2006) and the UEFA (Hägglund, Waldén, Bahr, & Ekstrand, 2005). The training will be conducted in a youth team that will not participate in the main study. Discussions and consultations among the research team members will be organized to ensure common understanding and performance of the measurement and recordings. They will also be trained on the standardized way of communicating with coaches and medical personnel regarding questions that may be asked.

Coaches and assistant coaches of the teams in the first and second division will be approached by the researcher where they will be explained the aims, objectives, rationale and procedure for the study. Further appointments will be organized for more explanation and opportunity to ask any questions pertaining to the study. Verbal explanations will be provided to the coaches in addition to the participant information sheet requesting them to participate in the study. Henceforth written consent will be sought from the coaches signing the consent form. This will be done during the preseason period.

The coaches will be requested to allocate time that will be convenient for them and the players to perform the measurement and test pertaining to the study.

Player exposure forms will then be provided to the coaches and will be requested to record every training time that each player participate in soccer activities. Because coaches are focusing on the game during the match, it could be hectic for them to record match exposure. For this reason, match exposure will be obtained from the reports that are submitted by the referee to the FERWAFA.

Before the beginning of each season the FERWAFA set a deadline for the teams to submit the list of all the players that they will use in the league. Therefore the list of all the players will be obtained from the FERWAFA while additional information pertaining to the study will be requested form the team management.

All the medical personnel of the teams will be invited into the briefing meeting that will be held at the FERWAFA premises to explain to them about the study and their task in the study. They will be explained how to fill the injury report forms and they will be asked to report any injury that will take place during data collection. The injury report and exposure forms will be collected by the researcher or the research assistants after every two weeks.

Training and match exposure as well as injury recording for the main study will commence one week before the first game because training performed during that week is for the preparation of the first game. The first division season will start in September 2014 while the second division season will start in November 2014. The exact dates will be obtained from the FERWAFA after the general assembly meeting preparing the league that will take place after the season 2013-2014. Data collection will take place throughout the season up to the last game. Because the FERWAFA allows the teams to recruit new players during the mid season, players who will join the teams halfway through will be recorded from the period recruited. The season will end in June 2015 for the first division and May 2015 for the second division. At the end of the season, the last two teams from the first division are relegated to the second division while the first two teams in the second division are promoted to the first division. A period of rest is given to the

teams and players at the end of the season 2014-2015 which allows teams to reorganize and recruit new players. Changes that will take place during this period will be noted and appropriate actions will be done to allow the injury surveillance to continue in the subsequent season. The study two with intervention will commence at the beginning of the season 2015-2016.

Figure 2: Study one framework

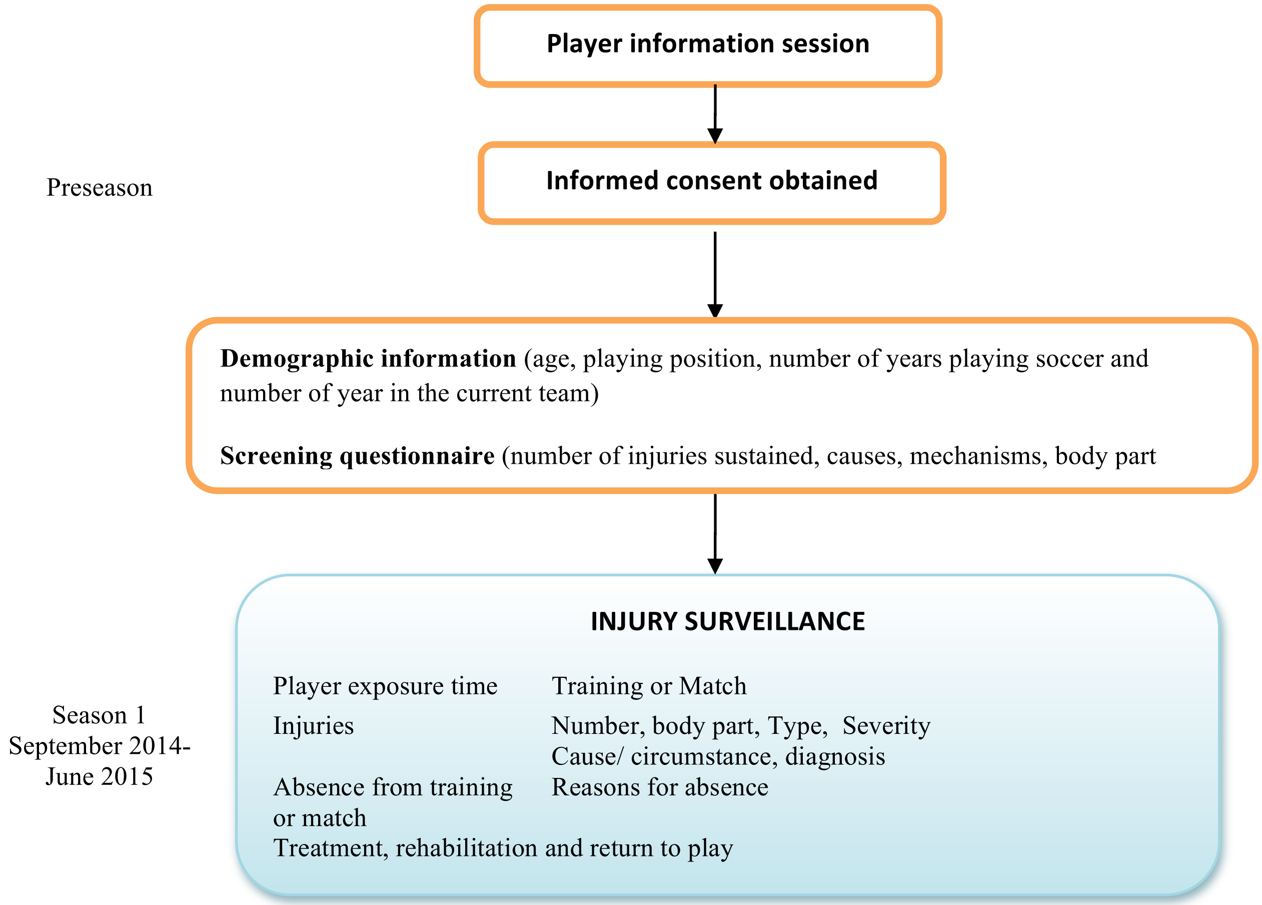

2.3.7 Pilot study

The pilot study will be conducted among coaches and medical personnel of the football academies that do not have teams that will participate in the main study. The pilot study will provide the opportunity to assess the use of the data collection forms, measurements and the

questionnaire. A meeting will be held with the participants to discuss about the instruments. The changes will be used to design more appropriate instruments.

2.3.8 Data management

After obtaining the list of all the players from the FERWAFA, they will be assigned codes that will be used to identify them for confidentiality. Collected forms will be kept in a locked and secure drawer in the researchers' office. Moreover, forms will be scanned to keep the soft copies of the collected data that will be saved on a password protected computer and external hard drive for back up.

2.3.9 Statistical analysis

All data will be analyzed statistically using STATISTICA version 12. Standard descriptive statistics will be used for numerical variables as means, standard deviations (SD) or 95% confidence intervals (CI).

In this study incidence for all injuries will be expressed as injuries per 1000 soccer playing hours for the purpose of the comparison with previous epidemiological studies. Risk per 1000 hours of exposure either per match, training or both will be equal to number of injuries multiplied by 1000 per total exposure time. The variations of injuries during match, training or both for the two seasons will be analyzed using Analysis of Variance (ANONA). ANOVA will also be used to analyze the occurrence of injuries according to the position in the team and division of play. The categorical variables such as the frequency, type, location, severity as well as the mechanism of injuries will be analyzed using a Chi-square test or Fisher's exact test for small numbers. Between groups incidence of injuries will be compared using z-statistics and rate ratios (RR) with a 95% CI will used to present data. The level of significance for all the tests is set to 0.05. Univariate and multivariate regression analyses will be used to calculate odds ratios (ORs) and 95% confidence intervals (CIs) for ±1 standard deviation of change. The details of all the statistical analysis that will be performed are provided in the appendix J.

2.3.10 Ethical considerations

Ethical approval to conduct the study will be obtained from the Faculty of Health Sciences Human Research Ethics Committee from the University of Cape Town. Permission to conduct the study will be sought from the Ministry of Sport and Culture and the FERWAFA as well as from the team leaders. The aim of the study will be explained to the coaches and players where they will be provided with the information to empower them to make informed choices about participation in the study. Written informed consent will be obtained from all the participants (Appendices F, G, H, I). The participants will be assured of confidentiality and anonymity. The data will be coded to maintain confidentiality, no names of the participants will be used but they will be assigned a numeric code. Only the researcher would have access to the collected raw data, and it will be kept in a safe locker. Participants will be explained their right to withdraw from the study at any stage without any implication. There is no obvious risk involved in participating in the study. All interested parties will have access to the findings of the study.

2.4STUDY TWO: INJURY PREVENTION INTERVENTION

2.4.1 Aim 1

One purpose of this study is to explore the difference in the coaches' knowledge, perception of, and readiness to accept a sports injury prevention programme.

2.4.1.1 Objectives

a. To assess if there is an increase in the knowledge of coaches towards injury prevention before and after the intervention
b. To assess if there is any difference in perception of the soccer coaches towards injury prevention before and after intervention
c. To assess whether the introduction of an injury prevention programme will increase the readiness for injury prevention practice of soccer coaches

2.4.2 Aim 2

A second purpose of the study is to assess the use of a warm up programme on the reduction of injuries in soccer.

2.4.2.1 Objectives

a. To assess if there is any difference in the occurrence of injuries before and after the introduction of the injury prevention warm up programme
b. To assess if the compliance of the injury prevention warm up programme will be associated with the reduction injuries.
c. To determine if there are any differences in injury incidence and exposure time between groups or over time

2.4.3 Study design

A cluster-randomized controlled trial will be used in this study with teams as units of randomization.

2.4.4 Participants

Coaches and assistant coaches of the teams in the second division teams will participate in this study. The coaches have been the center of focus in recent years as the main actors in the development of soccer in Rwanda. Much training has been organized by FIFA and CAF to enhance the knowledge and practice of the coaches. Trainings pertaining to technical, tactical and physical performance of the players were provided. At the end of the training, coaches are provided certificates. Not all coaches in Rwanda are citizens and not all of them had the same training. There are therefore some differences that may arise from their coaching training, background and experience.

The coaches are required to plan and supervise training sessions to develop the fitness and the skills of the payers. Coaches are responsible for the development of the training sessions according to the demands of the competition and the period of the season. The involvement of the coach in the first division teams concerning injuries in Rwanda is relatively less compared to the coaches in the second division because the teams have medical personnel who provide assistance. They also have assistant coaches, sometimes goalkeeper coaches, and kit managers. Coaches in the second division do not have all the support needed for their work. Working with

young players, they are required to act as organizers, parents, teachers, counselors, discipliners, for them to achieve results. In the case of injuries, coaches are looked at as mature and responsible people to provide first aid, preliminary management and advice for treatment. Coaches are further responsible to allow the players to return to play after injuries.

There are 19 teams in the second division and each team has at least one head coach. The minimum number of coaches of the teams who will receive the training is 19. The assistant coaches and goalkeeper coaches of the teams in the intervention study will also participate in the study even though not all the teams have them. The total number of players participating in the in the second division league is 570.

2.4.4.1 Inclusion criteria

Head coaches and their assistants as well as goalkeeper coaches of the teams participating in the second division league will be included in this study. Players who have contracts and licenses of teams enrolled to play in the second division will be included in the study. Players presenting injury at the beginning of the study will be included when cleared to join teams' training and matches. Players with injuries at the start of the study will be included, but the pre-existing injuries will not be included in data analysis. Players or teams that will leave or join the league before the end of the study will be included in the analysis for their time of participation.

2.4.4.2 Exclusion criteria

Players and coaches who will not agree to participate in the study will be excluded from the study.

2.4.4.3 Sample size / power analysis

Based on the study conducted by Twizere (2004), about 70% of soccer players in Rwanda sustained injuries. To obtain a 20% reduction of injuries with the implementation of the 11+ programme with the power of 90% and alpha of 5%, a sample size of 125 in each group (intervention and control) will be required. Taking into consideration the design effect of 2 for cluster randomization, the minimum number of players that should participate in this study is 217.

2.4.4.4 Recruitment and Sampling method

As there are 19 teams participating in the second division league, a random sampling method was used to obtain the group with nine or ten teams. Random sampling technique will be done to allocate the teams into the groups. Because of the promotion of the first two teams in the first division and the relegation of the last two teams into the second division, the teams will be selected towards the beginning of the season 2014-2015.

2.4.4.5 Blinding

The teams are organized in such a way that coaches are the ones planning all the activities to be performed by the players. In this study they are the ones responsible for implementing the intervention programme. Therefore, it is not possible to blind the coaches or the players to the allocation of the teams in either the intervention or the control group. The researcher will not be blinded to team allocation but the research assistants will be blinded to team allocation as each assistant will be assigned to one team without any information about whether the team is in the intervention or control group. Research assistants will not know the type of intervention provided to the coaches.

2.4.5 Pre- and post-tests

2.4.5.1 Knowledge assessment questionnaire

Questionnaires will be distributed to the coaches to assess their knowledge and belief related to injury prevention. Furthermore, coaches' readiness for injury prevention practice will be assessed prior to the intervention (Appendix K). The questionnaire will be comprised of 4

sections: section A will assess the demographic characteristics of the coaches including the age, highest coaching qualification, highest academic qualification, number of years coaching, number of years coaching first/ second division teams and number of years in the current team. In this section, they will also be asked whether they have received any first aid or injury prevention training. Section B assesses availability of the medical care in the team. Section C assesses injury prevention belief and practice readiness in key areas like planning injury prevention practice, use of protective equipment, checking playing field and facilities and up to date information. Section D assesses the first aid knowledge.

The validity and reliability of the questionnaire was established in a study conducted among high school coaches. The validity of the questionnaire was established during the two stage Delphi methods while the test-retest reliability was done to ascertain its reliability. Constructs pertaining to the stage of change, perceived susceptibility, benefits and self-efficacy were all Cronbach's alpha 70% and above (Siwon, 2013).

Looking at the fact that the knowledge constructs yielded a 39% Cronbach's alpha. An additional questionnaire used to assess the first aid and injury prevention knowledge was added to the previous one. The questions were developed by the American Red Cross after completion of first aid and sports injury courses to assess the gained knowledge. Participants were required to score minimum 80% to obtain certificates.

The questionnaires originally written in English will be translated in Kinyarwanda. Kinyarwanda is the national language which is spoken by all the Rwandan people. The questionnaire will be translated into Kinyarwanda by the researcher and the expert from the Rwanda Red Cross. The Kinyarwanda questionnaire will then be back-translated into English by a professional translator. It will then be piloted among five coaches in schools. The consensus meeting will be held to discuss the divergent areas in the two versions.

2.4.6 Intervention

Coaches of the teams in the second division in the intervention group will be invited to attend the training. The intervention is comprised of a five-day training divided into two parts. The first part of the training is the “11+” injury prevention programme while the second part is the training on first aid.

The “11+” injury prevention programme

The intervention programme “The 11+” will be introduced to coaches during the training (Appendix L). Coaches will be instructed on the 15 evidence-based exercises and the promotion of Fair Play. They will be instructed on how the exercises focus on core stability, balance, dynamic stabilization, and eccentric hamstring strength and their effects on frequent injury prevention. On field practical demonstration will also be conducted. At the end of the training, each coach will receive a DVD of the exercises and the total programme instructions and a booklet for reference. Coaches will be instructed to use the training programme at least 3 times a week and at every match and will record dates and times of using the training programme. Coaches will be visited at team practice by the researcher two times a week to see if proper implementation is occurring and additional instructions will be provided where necessary. The intervention will be conducted during the preseason period, October 2015.

First aid training

The intervention is comprised of 2-day training where they will be trained on first aid measures. Coaches will be trained on acute on-field, airway and concussion injuries management (Appendix M). A brief anatomy of some structures of the body (ankle, knee, shoulder, hamstrings, spine, and head) will be taught to the coaches (www.boksmart.co.za). Quick on field assessment of the injured player and guidelines on when to remove a player from the field will also be provided using the SALTAPS and PRICE guidelines developed by F-MARC (www.fifa.com).

2.4.7 Procedure

2.4.7.1 Data collection

Ethical approval, permissions and consent would be obtained during season one before conducting the study. Translation of the questionnaires into Kinyarwanda will be done before the pilot study. The translated questionnaires will be piloted among licensed five soccer coaches at school level to assess for clarity and responsiveness. A meeting will be held with them to discuss any unclear question from the translated versions and possibilities for better refining them.

During the preseason period, questionnaires assessing the knowledge of coaches regarding injury prevention will be distributed to coaches and appointment will be made for collection of the filled questionnaire. Questionnaires will be collected either by the researcher or the research assistants. Information pertaining to the availability of the medical personnel will also be obtained from the questionnaires.

Because of the changes that might take place at the end of the first season, coaches will again be invited to answer the knowledge questionnaires that will be distributed to them during the preseason period. Any other changes that will take place will also be noticed before the start of the season 2015-2016 as to the arrival of the new or change of the coaches, new teams registering into the second division league or new medical personnel arriving into the teams.

During the preseason period, July-August 2015, all the head, assistants and goalkeeper coaches of the teams in the intervention group in the second division will be invited to the training. The first part of the training will be conducted in two days where they will be taught about injury prevention in general and more specifically the importance and the use of the "11+" warm up programme. The first day will be for introduction and theoretical explanation of the conduct of the "11+" exercises while the second day will be reserved for practical demonstrations. The training will be provided by the researcher. Coaches will also be introduced to the recording of the use of the exercises while in the teams. The recording will be emphasized to ensure compliance. At the end of the training, the coaches will be provided DVDs, pamphlets and the FIFA link (where they could download the materials for free) of the exercises and will be

requested to use the “11+” as the warm up programme at least three times a week and before each match that the team will play.

Coaches will also be trained on first aid. During the training, they will receive the first aid course for two days that will be provided by the trainer from the Rwanda Red Cross. Questionnaires will be distributed to the coaches to assess their knowledge immediately after first aid training. At the end of season two (June 2016) questionnaires will be distributed to the coaches from both divisions to assess the knowledge retained after one year of intervention.

Coaches and medical personnel from the first division will be requested to continue the recording of injuries and exposure using the procedure they used in the season one (2014-2015). To increase compliance and proper recording, coaches will be visited at team practice by the researcher to see if proper implementation is occurring and additional instructions on the correct conduct of the exercises will be provided where necessary. Data collection during season two (2015-2016) will be the same as season one where players exposure time, occurrence of injuries, treatment will be assessed.

Figure 3: Study two framework

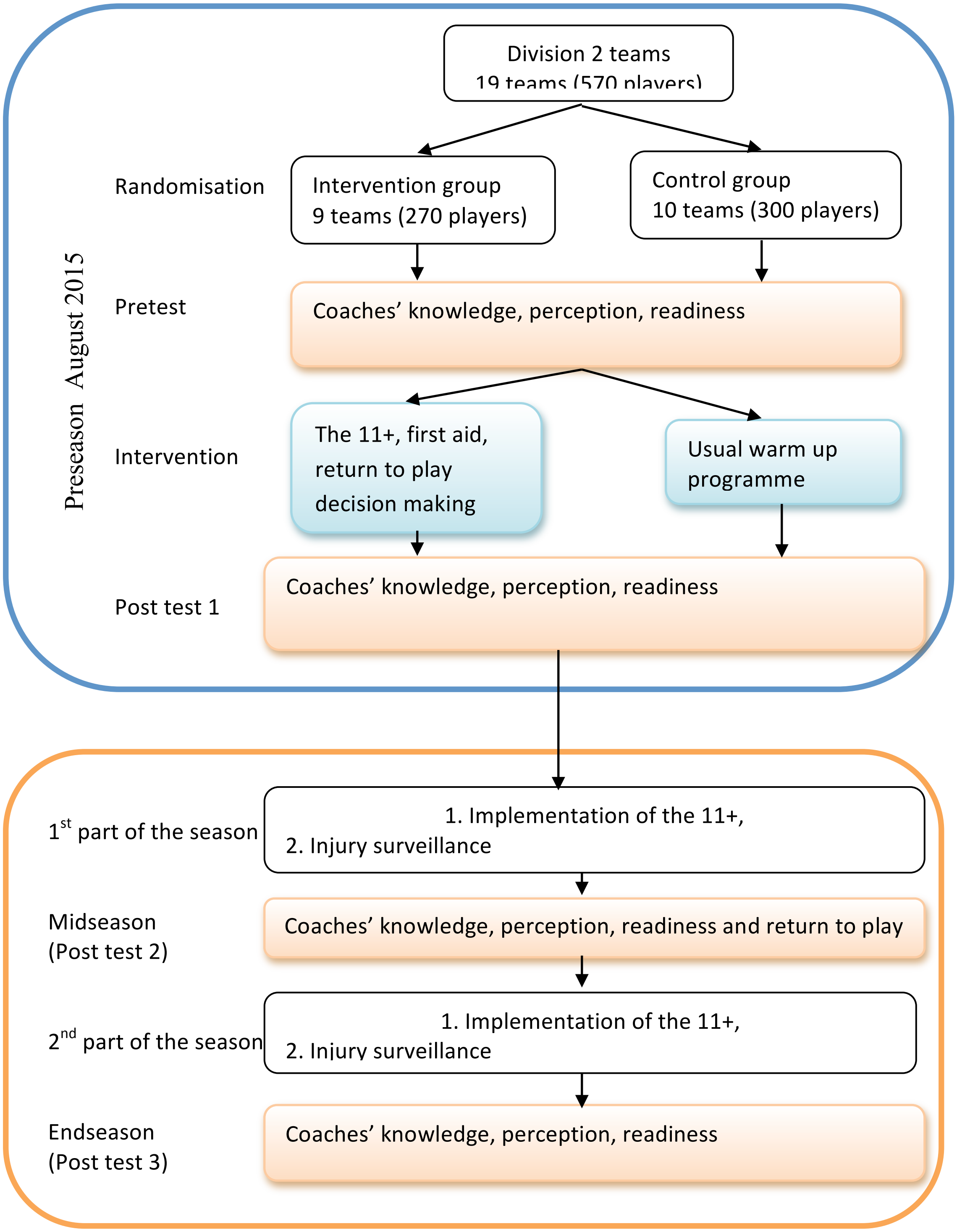

2.4.7.2 Compliance

Compliance with the programme will be assessed by providing to the coaches with additional forms to record the details of each time the "11+" will be used as the warm up into the team as well as the number of players who performed them (Appendix N). Completeness of the forms will be reviewed regularly by the research team.

2.4.7.3 Data management

The hard copies of the forms from the coaches and the medical personnel will be kept in a locked and secure drawer in the researchers' office after being scanned to keep the soft copies of the collected data. The electronic copies will then be saved on a password protected computer and external hard drive for back up.

2.4.8 Statistical analysis

Analysis of the incidence of injuries comparing the two study groups and the two divisions will be done as explained in the study one. Demographic characteristics of the coaches and responses on the knowledge, perception, readiness and decision making assessment will be analysed using descriptive statistics (range, mean, standard deviation). Categorical data like the coaches' level of education, highest level of coaching license, availability of the medical personnel into the team will be presented as frequency and percentages. The five injury prevention readiness to practice questions will be coded from zero to five. The minimum score will be zero and the maximum score will be 25. The responses from the perception questions that are on a 5-point Likert scale will be recorded on a range from 1="very unlikely" to 5="very likely". Therefore the results will be analysed using the descriptive statistics with means and standard deviation. Pearson correlation will be used to assess the relationship between perception variable and coaches' readiness of sports injury prevention. The same test will be used to assess the association between readiness, perception, and first aid knowledge and compliance with the injury prevention programme. Analysis of variance test (ANOVA) will be used to analyse the difference between the coaches' perceptions, knowledge, and readiness regarding injury prevention in the first division and the second division. The level of significance for all the tests

is set to 0.05. The details of all the statistical analysis that will be performed are provided in the appendix J.

2.4.9 Ethical considerations

There is no special ethical approval, permissions or informed consent that will be required to conduct this study. Players and coaches of the teams in the intervention group will be informed that they would receive injury prevention warm up programme while the teams in the control group would continue their warm up as usual.

For coaches, there is no risk involved in participating in the study. For players, there is a risk of sustaining an injury, but the risk is minimal because the injury prevention programme and few physical tests requires players to perform physical exercises under close supervision and at minimal to moderate intensity. Because players may be found into their teams, verbal information will be provided to them where the researcher will read them the information sheet. They will therefore be requested to sign the consent forms indicating their agreement to participate in the study.

REFERENCES

Alentorn-Geli, E., Mendiguchía, J., Samuelsson, K., Musahl, V., Karlsson, J., Cugat, R., & Myer, G. D. (2014). Prevention of anterior cruciate ligament injuries in sports-Part I: Systematic review of risk factors in male athletes. *Knee Surgery, Sports Traumatology, Arthroscopy : Official Journal of the ESSKA*, *22*(1), 3–15. http://doi.org/10.1007/s00167-013-2725-3

Andersen, J. C., Courson, R. W., Kleiner, D. M., & McLoda, T. A. (2002). National Athletic Trainers' Association position statement: Emergency planning in athletics. *Journal of Athletic Training*.

Arnason, a., Andersen, T. E., Holme, I., Engebretsen, L., & Bahr, R. (2008). Prevention of hamstring strains in elite soccer: An intervention study. *Scandinavian Journal of Medicine and Science in Sports*, *18*(1), 40–48. http://doi.org/10.1111/j.1600-0838.2006.00634.x

Bahr, R., & Krosshaug, T. (2005). Understanding injury mechanisms: a key component of preventing injuries in sport. *British Journal of Sports Medicine*, *39*(6), 324–329. http://doi.org/10.1136/bjsm.2005.018341

Brumitt, J., Heiderscheit, B. C., Manske, R. C., Niemuth, P. E., Rauh, M. J., McIntosh, L., … Rutt, R. (2013). Lower Extremity Functional Tests and Risk of Injury in Division Iii Collegiate Athletes. *The International Journal of Sports Physical Therapy*, *8*(3), 216–227.

Daneshjoo, A., Mokhtar, A. H., Rahnama, N., & Yusof, A. (2013). the Effects of Injury Prevention Warm-Up Programmes on Knee Strength in Male Soccer Players. *Biology of Sport*, *30*(4), 281–288. http://doi.org/10.5604/20831862.1077554

De Freitas Guina Fachina, R. J., Andrade, M. D. S., Silva, F. R., Waszczuk-Junior, S., Montagner, P. C., Borin, J. P., & de Lira, C. A. B. (2013). Descriptive epidemiology of injuries in a Brazilian premier league soccer team. *Open Access Journal of Sports Medicine*, *4*, 171–4. http://doi.org/10.2147/OAJSM.S44384

Dennis, R. J., Finch, C. F., Elliott, B. C., & Farhart, P. J. (2008). The reliability of musculoskeletal screening tests used in cricket. *Physical Therapy in Sport*, *9*(1), 25–33. http://doi.org/10.1016/j.ptsp.2007.09.004

Dennis, R. J., Finch, C. F., McIntosh, a S., & Elliott, B. C. (2008). Use of field-based tests to identify risk factors for injury to fast bowlers in cricket. *British Journal of Sports Medicine*, *42*(6), 477–482. http://doi.org/10.1136/bjsm.2008.046698

Ekstrand, J., Hägglund, M., & Waldén, M. (2011). Injury incidence and injury patterns in professional football: the UEFA injury study. *British Journal of Sports Medicine*, *45*(7), 553–8. http://doi.org/10.1136/bjsm.2009.060582

Finch, C. (2006). A new framework for research leading to sports injury prevention. *Journal of Science and Medicine in Sport*, *9*(1-2), 3–9. http://doi.org/10.1016/j.jsams.2006.02.009

Fuller, C. W., Ekstrand, J., Junge, a, Andersen, T. E., Bahr, R., Dvorak, J., … Meeuwisse, W. H. (2006). Consensus statement on injury definitions and data collection procedures in studies of football (soccer) injuries. *British Journal of Sports Medicine*, *40*(3), 193–201. http://doi.org/10.1136/bjsm.2005.025270

Fuller, C. W., Junge, A., & Dvorak, J. (2012). Risk management: FIFA's approach for protecting the health of football players. *British Journal of Sports Medicine*, *46*(1), 11–7. http://doi.org/10.1136/bjsports-2011-090634

Gabbe, B. J., Bennell, K. L., Wajswelner, H., & Finch, C. F. (2004). Reliability of common lower extremity musculoskeletal screening tests. *Physical Therapy in Sport*, *5*(2), 90–97. http://doi.org/10.1016/j.ptsp.2004.01.003

Gaskell, L. (2013). Musculoskeletal assessment. *Tidy's Physiotherapy: Fifteenth Edition*, 207–251. http://doi.org/10.1016/B978-0-7020-4344-4.00011-0

Gribble, P. a., Hertel, J., & Plisky, P. (2012). Using the star excursion balance test to assess dynamic postural-control deficits and outcomes in lower extremity injury: A literature and systematic review. *Journal of Athletic Training*, *47*(3), 339–357. http://doi.org/10.4085/1062-6050-47.3.08

Hägglund, M., Waldén, M., Bahr, R., & Ekstrand, J. (2005). Methods for epidemiological study of injuries to professional football players: developing the UEFA model. *British Journal of Sports Medicine*, *39*(6), 340–346. http://doi.org/10.1136/bjsm.2005.018267

Hägglund, M., Waldén, M., & Ekstrand, J. (2006). Previous injury as a risk factor for injury in elite football: a prospective study over two consecutive seasons. *British Journal of Sports Medicine*, *40*(9), 767–772. http://doi.org/10.1136/bjsm.2006.026609

Hägglund, M., Waldén, M., & Ekstrand, J. (2007). Lower reinjury rate with a coach-controlled rehabilitation program in amateur male soccer: a randomized controlled trial. *The American Journal of Sports Medicine*, *35*(9), 1433–1442. http://doi.org/10.1177/0363546507300063

Henke, T., & Luig, P. (2012). Safety in Sports. *Ruhr University Bochum. Sports Medicine*.

Junge, a. (2004). Football Injuries During FIFA Tournaments and the Olympic Games, 1998-2001: Development and Implementation of an Injury-Reporting System. *American Journal of Sports Medicine*, *32*(90010), 80S–89. http://doi.org/10.1177/0363546503261245

Junge, A., & Dvorak, J. (2004). Soccer injuries: A review on incidence and prevention. *Sports Medicine*. http://doi.org/10.2165/00007256-200434130-00004

Junge, A., Lamprecht, M., Stamm, H., Hasler, H., Bizzini, M., Tschopp, M., … Dvorak, J. (2011). Countrywide campaign to prevent soccer injuries in Swiss amateur players. *The American Journal of Sports Medicine*, *39*(1), 57–63. http://doi.org/10.1177/0363546510377424

Kirkendall, D. T., Junge, A., & Dvorak, J. (2010). Prevention of football injuries. *Asian Journal of Sports Medicine*, *1*(2), 81–92. http://doi.org/10.1007/88-470-0419-5_6

Kristenson, K., Bjørneboe, J., Waldén, M., Andersen, T. E., Ekstrand, J., & Hägglund, M. (2013). The Nordic Football Injury Audit: higher injury rates for professional football clubs with third-generation artificial turf at their home venue. *British Journal of Sports Medicine*, *47*(12), 775–81. http://doi.org/10.1136/bjsports-2013-092266

Luciano, A., & Lara, L. (2012). EPIDEMIOLOGICAL STUDY OF FOOT AND ANKLE. *Acta Ortopédica Brasileira Journal*, *20*(6), 339–342.

Maffey, L., & Emery, C. (2006). Physiotherapist delivered preparticipation examination: rationale and evidence. *North American Journal of Sports Physical Therapy : NAJSPT*, *1*(4), 176–186.

Mandelbaum, B. R., Silvers, H. J., Watanabe, D. S., Knarr, J. F., Thomas, S. D., Griffin, L. Y., … Garrett, W. (2005). Effectiveness of a neuromuscular and proprioceptive training program in preventing anterior cruciate ligament injuries in female athletes: 2-year follow-up. *The American Journal of Sports Medicine*, *33*(7), 1003–1010. http://doi.org/10.1177/0363546504272261

Nilstad, A., Andersen, T. E., Bahr, R., Holme, I., & Steffen, K. (2014). Risk factors for lower extremity injuries in elite female soccer players. *The American Journal of Sports Medicine*, *42*(4), 940–8. http://doi.org/10.1177/0363546513518741

Niyonsenga, J. D., & Phillips, J. S. (2013). Factors associated with injuries among first-division Rwandan female soccer players. *African Health Sciences*, *13*(4), 1021–1026. http://doi.org/10.4314/ahs.v13i4.23

Orchard, J. W., & Finch, C. F. (2002). Australia needs to follow New Zealand ' s lead on sports injuries. *Medical Journal of Australia*, *177*, 38–39.

Parry, L., & Drust, B. (2006). Is injury the major cause of elite soccer players being unavailable to train and play during the competitive season? *Physical Therapy in Sport*, *7*(2), 58–64. http://doi.org/10.1016/j.ptsp.2006.03.003

Sharkey, & Gaskill. (2006). Sports Physiology for Coaches.

Soligard, T., Myklebust, G., Steffen, K., Holme, I., Silvers, H., Bizzini, M., … Andersen, T. E. (2008). Comprehensive warm-up programme to prevent injuries in young female

footballers: cluster randomised controlled trial. *BMJ (Clinical Research Ed.)*, *337*(December), a2469. http://doi.org/10.1136/bmj.a2469

Soligard, T., Nilstad, A., Steffen, K., Myklebust, G., Holme, I., Dvorak, J., … Andersen, T. E. (2010). Compliance with a comprehensive warm-up programme to prevent injuries in youth football. *British Journal of Sports Medicine*, *44*(11), 787–93. http://doi.org/10.1136/bjsm.2009.070672

Steffen, K., Emery, C. a, Romiti, M., Kang, J., Bizzini, M., Dvorak, J., … Meeuwisse, W. H. (2013). High adherence to a neuromuscular injury prevention programme (FIFA 11+) improves functional balance and reduces injury risk in Canadian youth female football players: a cluster randomised trial. *British Journal of Sports Medicine*, *47*(12), 794–802. http://doi.org/10.1136/bjsports-2012-091886

Steffen, K., Myklebust, G., Olsen, O. E., Holme, I., & Bahr, R. (2008). Preventing injuries in female youth football--a cluster-randomized controlled trial. *Scandinavian Journal of Medicine & Science in Sports*, *18*(5), 605–14. http://doi.org/10.1111/j.1600-0838.2007.00703.x

Twizere, J. (2004). Epidemiology of Soccer injuries in Rwanda: A need for Physiotherapy intervention. *Cape Town, University of the Western Cape*, 154.

Van Beijsterveldt, A. M. C., van de Port, I. G. L., Krist, M. R., Schmikli, S. L., Stubbe, J. H., Frederiks, J. E., & Backx, F. J. G. (2012). Effectiveness of an injury prevention programme for adult male amateur soccer players: a cluster-randomised controlled trial. *British Journal of Sports Medicine*, *46*(16), 1114–8. http://doi.org/10.1136/bjsports-2012-091277

Van Mechelen, W., Hlobil, H., & Kemper, H. C. (1992). Incidence, severity, aetiology and prevention of sports injuries. A review of concepts. *Sports Medicine (Auckland, N.Z.)*, *14*(2), 82–99. http://doi.org/10.2165/00007256-199214020-00002

Verhagen, E. a L. M., van Stralen, M. M., & van Mechelen, W. (2010). Behaviour, the key factor for sports injury prevention. *Sports Medicine (Auckland, N.Z.)*, *40*(11), 899–906. http://doi.org/10.2165/11536890-000000000-00000

Appendix A : LIST OF PLAYERS' FORM

N°	Names	DOB	Playing position	Number of years playing football	Number of years in 1st/ 2nd division	Number of years in current club
1						
2						
3						
4						
5						
6						
7						
8						
9						
10						
11						
12						
13						
14						
15						
16						
17						
18						
19						
20						
21						
22						
23						
24						
25						
26						
27						
28						
29						
30						

Appendix B: MEDICAL RECORD FORM

Team Code:……………………………….

Player’s name:…………………………….

Player’s code: …………………………….

1. Have you sustained any injury during training or competitive match that have resulted in you missing the following session(s)?

   Training Injury ☐ How many ☐

   Match injury ☐ How many ☐

2. Which body parts sustained injury? (One or more answers are possible).

   Head: □ Face: □ Neck: □ Shoulder: □ Back: □ Chest: □

   Upper arm: □ Elbow: □ Fore arm: □ Wrist: □ Hand: □ Finger: □

   Pelvis: □ Buttock: □ Groin: □ Hip: □ Front thigh: □ Back thigh: □

   Knee: □ Shin:□ Calf: □ Ankle: □ Foot: □ Toe: □
