# Supplementary material for: Effect of the FIFA 11+ soccer specific warm up programme on the incidence of injuries: A cluster-randomised controlled trial
Source: PLoS One. 2021 May 24;16(5):e0251839. doi: 10.1371/journal.pone.0251839 (PMC8143390; doi:10.1371/journal.pone.0251839)
Supplement: S1 Appendix — (DOCX) [file pone.0251839.s004.docx]

Instructions on the type of exercise, important points to observe with repetitions and time as described in the FIFA Manual.

| *Part 1: Running exercises* | | | |
| --- | --- | --- | --- |
| *1* | *Straight ahead* | *Jog straight to the last cone. Run slightly more quickly on the way back. Make sure you keep your upper body straight. Your hips, knees and feet should be aligned. Do not let your knees buckle inwards.* | *Repeat 2 times* |
| *2* | *Hip out* | *Jog to the first cone. Stop and lift your knee forwards. Rotate your knee to the side and put your foot down. Jog to the cone and do the exercise on the other leg. When you have finished the course, jog back. Make sure that you keep your pelvis horizontal and your core still. The hip, knee and foot of the supporting leg should be aligned. Do not let the knee of the supporting leg buckle inwards.* | *Repeat 2 times* |
| *3* | *Hip in* | *Jog to the first cone. Stop and lift your knee to the side. Rotate your knee forwards and put your foot down. Jog to the next cone and do the exercise on the other leg. When you have finished the course, jog back. Make sure that you keep your pelvis horizontal and your core still. The hip, knee and foot of the supporting leg should be aligned. Do not let the knee of the supporting leg buckle inwards.* | *Repeat 2 times* |
| *4* | *Circling partner* | *jog forwards to the first cone. Shuffle sideways at a 90-degree angle towards your partner, shuffle an entire circle around one other (without changing the direction you are looking in) and back to the first cone.jog to the next cone and repeat the exercise. When you have finished the course, jog back. Bend your hips and knees slightly and carry your body weight on the balls of your feet. Do not let your knees buckle inwards.* | *Repeat 2 times* |
| *5* | *Jumping with shoulder contacts* | *jog to the first cone. Shuffle sideways at a 90-degree angle towards your partner. In the middle, jump sideways towards each other to make shoulder-to-shoulder contact. Shuffle back to the first cone. Then jog to the next cone and repeat the exercise. When you have finished the course, jog back. Land on both feet with your hips and knees bent. Do not let your knees buckle inwards.* | *Repeat 2 times* |
| *6* | *Quick forwards and backwards sprints* | *Run quickly to the second cone then run backwards quickly to the first cone, keeping your hips and knees slightly bent. Repeat, running two cones forwards and one cone backwards. When you have finished the course, jog back. Make sure you keep your upper body straight. Your hips, knees and feet should be aligned. Do not let your knees buckle inwards.* | *Repeat 2 times* |
| *Part 2: Strength, plyometrics and balance exercises* | | | |
| *7* | *The plank with one leg lift and hold* | ***Starting position:*** *Lie on your front, supporting yourself on your forearms and feet. Your elbows should be directly under your shoulders.*  ***Exercise:*** *Lift your body up, supported on your forearms, and pull your stomach in. Lift one leg about 10-15 centimetres off the ground, and hold the position for 20-30 sec. Your body should be straight. Do not let your opposite hip dip down and do not sway or arch your lower back. Take a short break, change legs* | *3 sets (20 – 30 sec. each)* |
| *8* | *Sideways bench with leg lift* | ***Starting position:*** *Lie on your side with both legs straight. Lean on your forearm and the side of your foot so that your body is in a straight line from shoulder to foot. The elbow of your supporting arm should be directly beneath your shoulder.*  ***Exercise:*** *Lift your uppermost leg up and slowly lower it down again. Repeat for 20-30 sec. Take a short break, change sides and repeat. Do not rest your head on your shoulder. Keep your pelvis stable and do not let it tilt downwards. Do not tilt your shoulders, pelvis or legs forwards or backwards.* | *3 sets (20 – 30 sec. on each side)* |
| *9* | *Hamstrings (Advanced)* | ***Starting position:*** *Kneel on a soft surface. Ask your partner to hold your ankles down firmly.*  ***Exercise:*** *Your body should be completely straight from the shoulder to the knee throughout the exercise. Lean forward as far as you can, controlling the movement with your hamstrings and your gluteal muscles. When you can no longer hold the position, gently take your weight on your hands, falling into a push-up position. Do not tilt your head backwards. Do not bend at your hips* | *1 set (minimum 12-15 repetitions) and/or 60 sec.* |
| *10* | *Single-leg stance (Testing partner)* | ***Starting position:*** *Stand on one leg opposite your partner and at arm’s’ length apart.*  ***Exercise:*** *Whilst you both try to keep your balance, each of you in turn tries to push the other off balance in different directions. Try to keep your weight on the ball of your foot and prevent your knee from buckling inwards. Continue for 30 sec. Do not let your knee buckle inwards. Do not let your pelvis tilt to the side.* | *2 sets (30 sec. on each leg)* |
| *11* | *One-leg squats* | ***Starting position:*** *Stand on one leg, loosely holding onto your partner.*  ***Exercise:*** *Slowly bend your knee as far as you can manage. Concentrate on preventing the knee from buckling inwards. Bend your knee slowly then straighten it slightly more quickly, keeping your hips and upper body in line. Do not let your knee buckle inwards. Your bent knee should not extend beyond your toes. Do not twist or tilt your pelvis to the side.* | *2 sets (10 on each side)* |
| *12* | *Box Jumps* | ***Starting position:*** *Stand with your feet hip-width apart. Imagine that there is a cross marked on the ground and you are standing in the middle of it.*  ***Exercise:*** *Alternate between jumping forwards and backwards, from side to side, and diagonally across the cross. Jump as quickly and explosively as possible. Your knees and hips should be slightly bent. Land softly on the balls of your feet. Do not let your knees buckle inwards. Do not let your knees buckle inwards. Do not land with extended knees or on your heels.* | *2 sets (30 sec.)* |
| *Part 3: Running exercises* | | | |
| *13* | *Across the pitch* | *Run approximately 40 metres across the pitch at 75 – 80% of maximum pace and then jog the rest of the way.jog back at an easy pace. Make sure you keep your upper body straight.*  *Your hips, knees and feet should be aligned. Do not let your knees buckle inwards.* | *Do the exercise twice.* |
| *14* | *Bounding* | *Take a few warm-up steps then take 6 – 8 bounding steps with a high knee lift and jog the rest of the way. With each bound, try to lift the knee of the leading leg as high as possible and swing the opposite arm across the body. Jog back at an easy pace to recover.*  *Keep your upper body straight. Land on the ball of the leading foot with the knee bent and spring. Do not let your knee buckle inwards.* | *Do the exercise twice.* |
| *15* | *Plant and cut* | *Jog four to five steps straight ahead. Then plant on the right leg and cut to change direction to the left and accelerate again. Sprint for 5 – 7 steps (at 80 – 90% of maximum pace) before you decelerate and plant on the left foot and cut to change direction to the right. Repeat the exercise until you reach the other side of the pitch, then jog back.*  *Make sure you keep your upper body straight. Your hips, knees and feet should be aligned. Do not let your knees buckle inwards.* | *Do the exercise twice.* |

Available at https://www.fifamedicalnetwork.com/wp-content/uploads/cdn/11plus_workbook_e.pdf
